# Below-ground competition alters attractiveness of an insect-pollinated plant to pollinators

**DOI:** 10.1093/aobpla/plaa022

**Published:** 2020-06-12

**Authors:** Floriane Flacher, Xavier Raynaud, Amandine Hansart, Benoît Geslin, Eric Motard, Séléné Verstraet, Manon Bataille, Isabelle Dajoz

**Affiliations:** 1 Sorbonne Université, CNRS, IRD, INRAE, Université de Paris, UPEC, Institute of Ecology and Environmental Sciences-Paris, Paris, France; 2 Aix Marseille Univ, Avignon Université, CNRS, IRD, IMBE, Marseille, France; 3 Centre de recherche en écologie expérimentale et prédictive (CEREEP-Ecotron IleDeFrance), Département de biologie, Ecole normale supérieure, CNRS, PSL University, St-Pierre-les-Nemours, France

**Keywords:** Below-ground competition, floral display size, flower visitors, *Holcus lanatus*, pollination, *Sinapis alba*

## Abstract

Competitive interactions between plants can affect patterns of allocation to reproductive structures through modulation of resource availability. As floral traits involved in plant attractiveness to pollinators can be sensitive to these resources, competition with any neighbouring species may influence the attractiveness of insect-pollinated plants. While pollination research has primarily focused on above-ground interactions, this study aims at investigating if the presence of a competitor plant can modulate neighbouring insect-pollinated plant attractiveness to pollinators and resulting fecundity, especially through below-ground competitive interactions for soil resources. We set up a plot experiment in which we grew an insect-pollinated plant, *Sinapis alba* (Brassicaceae), in a mixture dominated by a wind-pollinated plant, *Holcus lanatus* (Poaceae). Individuals of *S. alba* were either subjected to or isolated from (with buried tubes in the soil) below-ground competition. Across the flowering season, floral traits involved in attractiveness of *S. alba* and pollinator visitation were followed at the plot and plant level to investigate different scales of attractiveness. At the end of the experiment, seeds were harvested to assess plant fecundity. Competition had a significant negative effect on plot and plant floral display size as well as flower size while nectar traits were not affected. When plants of *S. alba* were in competition, the time to first visit was altered: the proportion of plots that received a visit was smaller for a given time; in other words, it took more time for a given proportion of plots to be visited and some plots were even never visited. Moreover, pollinators made fewer visits per plots. The proportion of viable seeds produced by *S. alba* in competition was lower and probably linked to the competition itself rather than changes in pollinator visitation. This study suggests that competitive interactions between plants can modulate pollination interactions even when competing plant species are not insect-pollinated.

## Teaser

Through a plot experiment, this study shows that the presence of a competitor plant can modulate neighbouring insect-pollinated plant attractiveness to pollinators through below-ground competition for soil resources. Several floral traits as well as pollinator visitations were negatively influenced in condition of competition. A decrease of plant fecundity was also observed but probably linked to the competition itself rather than changes in pollinator visitation induced by competition. Still, this study suggests that, in a plant mixture, the presence of any competitor plant may influence plant–pollinator interactions and future studies on pollination should benefit from considering the whole plant community.

## Introduction

Studying biotic interactions and their above-ground–below-ground relationships is considered a major topic in ecology (Bardgett 2018) especially when focusing on plants that are part of both compartments. As animal pollination is a key driver of plant community functioning (Ollerton *et al.* 2011), it is of major importance to investigate if below-ground plant–plant interactions can influence plant–pollinator relationships. Plant visitation depends on the production of visual or olfactory cues that attract pollinators. These cues (i.e. floral attractiveness traits) are generally related to flower features such as their number, size, colour or scent as well as associated rewards (e.g. pollen and nectar, Dafni 1992; Proctor *et al.* 1996; Willmer 2011). Plant attractiveness to pollinators can also operate at different scales, from long to short distance (Dafni 1992; Dafni *et al.* 1997), depending especially on the target plant’s local environment (e.g. neighbouring plants) and pollinators’ sensory acuity (e.g. the ability to discriminate a target plant from its background) (van der Kooi *et al.* 2019). For instance, pollinators may rely on flowered plant aggregation for long-distance detection (Dafni *et al.* 1997) while floral traits (e.g. plant floral display size, flower size) would influence pollinator preferences at shorter distances.

Floral attractiveness traits can be sensitive to environmental factors, especially variation in soil resource availability (e.g. nutrients, water). Many studies have shown an increase in flower production, bloom duration, flower size or floral display size with nutrient or litter input (Campbell and Halama 1993; Muñoz *et al.* 2005; Burkle and Irwin 2009, 2010; Hoover *et al.* 2012; Phoenix *et al.* 2012; Soper Gorden and Adler 2013) as well as increasing soil moisture (Caruso 2006; Halpern *et al.* 2010; Gallagher and Campbell 2017). Soil fertilization or water availability can also influence nectar quantity (Halpern *et al.* 2010; Waser and Price 2016; Gallagher and Campbell 2017) and quality, especially regarding sugar (Baude *et al.* 2011), amino acid (Gardener and Gilman 2001; Gijbels *et al.* 2015; Ceulemans *et al.* 2017) or alkaloid content (Adler *et al.* 2006). As they influence soil nutrient availability or plant nutrient uptake ability, below-ground biotic interactions can also modulate floral attractiveness traits. For instance, mycorrhizal fungi and N-fixing bacteria can positively influence floral display size, nectar production and modify nectar chemistry, likely through enhanced access to limiting nutrients or greater water use efficiency (see Barber and Soper Gorden 2014 for a review).

Likewise, when focusing on plant–pollinator interactions, competition between plants can affect floral parameters of insect-pollinated plants, such as floral display size, flower production or nectar quantity and quality (Baude *et al.* 2011; Partzsch and Bachmann 2011; Flacher *et al.* 2015, 2017). This is likely due to changes in plant access to nutrients as plant competition can influence their local availability (Berntson and Wayne 2000; Raynaud and Leadley 2004). Moreover, Flacher *et al.* (2015, 2017) found that the impact of competitive interactions between plants on attractiveness traits was stronger as the intensity of competition increased. At the plant community level, the attractiveness of an individual plant may depend on its own floral traits but also on floral traits of neighbouring plant individuals or species (Willmer 2011). Most pollinators tend to preferentially visit plants that have large floral displays (Goulson 2003; Mitchell *et al.* 2004), large flowers (Conner and Rush 1996) or greater reward, especially in quality (Cnaani *et al.* 2006). As a result, plant species that are identified as more attractive are more visited. Below-ground plant–plant interactions, that lead to a decrease in floral attractiveness traits, can thus have a negative influence on pollinator visits as well. Finally, because plant reproductive success can be closely linked to the foraging behaviour of pollinators (Cayenne Engel and Irwin 2003; Karron *et al.* 2006), we can assume that competition between plants for soil resources could yield a bottom-up effect on plant–pollinator interactions which could, in turn, influence the reproductive success of insect-pollinated species. To our knowledge, while bottom-up effects on plant–pollinator interactions have been studied for soil resource addition (Muñoz *et al.* 2005; Burkle and Irwin 2009) as well as mycorrhizal fungi and root herbivores (see Barber and Soper Gorden 2014 for a review), it has not been investigated yet for plant competition and deserves attention.

In this study we set up a field experiment in which an insect-pollinated plant (*Sinapis alba*) was grown in a mixture dominated by a wind-pollinated plant (*Holcus lanatus*). By choosing a wind-pollinated plant competitor, this study focuses on competition between plants for abiotic resources only, excluding competition for pollinators that would have influenced pollinator response to floral traits (Levin and Anderson 1970). *Sinapis alba* and *H. lanatus* were studied in experimental plots with or without below-ground competition. Our objectives were to study whether below-ground competition could affect (i) floral traits, (ii) pollinator visitation and (iii) plant fecundity. Especially, we wondered if plant attractiveness to pollinators was influenced both at long and short distance. To do so, we studied several floral traits and pollinator visitation variables at plot or plant level and whether they reflected long- or short-distance attractiveness. We expected below-ground competition to have a negative effect on pollinator visits (both at long and short distance) and subsequent plant fecundity through a negative effect on floral traits involved in attractiveness to pollinators.

## Materials and Methods

### Study system

#### Focal insect-pollinated species.

*Sinapis alba* (Brassicaceae) is an annual forb that grows along roads, in wastelands or near crops. This species is a CR strategist (i.e. a competitor–ruderal plant according to Grime’s life strategies; Grime 1977; Kühn *et al.* 2004) and is considered naturalized in the Ile-de-France region, where the experiment took place (Lombard 2001). *Sinapis alba* is an obligate outcrossing species (Olsson 1960). From May to July, *S. alba* produces yellow open flowers that can be visited by honeybees, bumblebees as well as shorter-tongued flower visitors like solitary bees and hoverflies (Pareja *et al.* 2012; Theodorou *et al.* 2016; Karamaouna *et al.* 2019). Fruits are siliques containing <8 seeds (Jauzein 2011).

#### Competition mixture.

*Holcus lanatus* is a wind-pollinated perennial grass that grows in grasslands, wastelands or along road and crops. It produces large amounts of biomass (C strategist, a competitor plant—Grime 1977; Kühn *et al.* 2004) and is known to negatively affect floral traits of insect-pollinated species through competitive interactions (Flacher *et al.* 2015). Because the initial design was built to focus on the response of two insect-pollinated plants to below-ground competition, each plot of the experiment also contained 12 individuals of *Echium* plants (initially, *E. plantagineum* but furnished seeds were from mislabelled seed batches of *E. vulgare*). All 12 plants were arranged in the same way than *S. alba* plants **[see****Supporting Information—Fig. S1****]***. Echium vulgare* is a biennial species (CR strategist) and none of the plants bloomed during the experiment. As *H. lanatus* is known to outcompete other *Echium* species (Flacher *et al.* 2015), we assumed that competitive interactions induced by this mixture were mostly due to the wind-pollinated plant (**see****Supporting Information—Fig. S1** for more information).

### Experimental set-up

The experiment took place in a grassland site (CEREEP Ecotron Ile-de-France, Saint Pierre lès Nemours, France) on sandy soil (pH = 6). In February 2014, the top 10 cm of soil were ploughed and steam-sterilized to remove the seed bank and existing vegetal material (e.g. plant stolons) that could interfere with experimental treatments. In March 2014, 10 experimental plots (0.9 × 0.9 m) were delimited and sown with 25 g of *H. lanatus* seeds per plots. In April 2014, 12 seedlings of *S. alba* were planted in each experimental plot along parallel **[see****Supporting Information—Fig. S1****]** for a total of 120 *S. alba* plants. Prior to planting seedlings, 12 PVC tubes (12.5 cm diameter, 30 cm deep) were buried in half of experimental plots to isolate *S. alba* roots from other plant roots (the ‘without competition’ treatment C−, treatment 2 in **Supporting Information—Fig. S1**). The five remaining plots contained plant of *S. alba* with roots subjected to below-ground competition (the ‘with competition’ treatment C+, treatment 4 in **Supporting Information—Fig. S1**). As soil moisture was not homogenous in the field, experimental plots were organized in blocks, each block containing both treatments. All plots were enclosed in a nylon mesh cage (2 × 2 × 2 m, 950-µm mesh) to prevent pollinators from uncontrolled visits on flowers outside of experimental surveys. Because these nylon mesh cages could cast shadows on the neighbouring cages, edge effects were observed on two experimental plots (one per treatment) which were discarded from the analysis, thus leading to 4 replicates per treatment. In the end, 8 experimental plots (2 treatments × 4 blocks or replicates) and 96 plants of *S. alba* (8 plots × 12 plants) were followed across the whole growing season. Plots were all watered when necessary. Non-target plants were removed manually. The effect of above-ground competition was limited in our experiment as (i) below-ground competition is more important than above-ground competition, especially when the competitor is a grass (Kiær *et al.* 2013) and (ii) shoots of all plants but *S. alba* were regularly cut (10–15 cm high, all experimental plots at the same time) to prevent above-ground competition.

### Plant height and biomass

At the beginning of the experiment, four individuals of *S. alba* were randomly chosen in every plot to follow their growth. This was estimated by measuring plant height (cm) from the ground surface to the apical meristem (the highest raceme when the plant was ramified) at days 8, 35 and 63 after the start of the experiment (i.e. 1 week and the first 2 months after planting). Final above-ground and below-ground biomass of all *S. alba* plants were harvested, oven dried (65 °C, 72 h) and weighed.

### Attractiveness traits of *S. alba*

On 55 days from 12 June to 11 September 2014, plant flower production (i.e. the number of newly open flowers) and plant floral display size (i.e. the number of open flowers, both fresh and old) were measured on all 96 plants. From the plant floral display size, we calculated a plot floral display size as the sum of open flowers on all 12 plants in each plot. On every plant, additional traits (flower size and nectar traits) were measured on a maximum of six flowers randomly selected on three plants per plot (two flowers at most per plant). This sampling was restricted because we made nectar trait measurements (i) on newly open flowers only, limiting the influence of flower age on variation in flower size and reward production (Southwick and Southwick 1983; Galetto *et al.* 1994) and (ii) before each behavioural observation to avoid nectar shortage due to pollinator foraging. No measurements were made if the number of newly open flowers was insufficient (i.e. <3 newly open flowers on the same plant) so that pollinators could have resource to seek. Flower size was measured with a digital calliper as the distance between the tips of two opposed petals. Nectar volume was estimated using calibrated microcapillary tubes (0.5 µL, 32 mm Minicaps end to end, Hirschmann Laborgeraete). Nectar sugar concentration was measured right after sampling on the field, using hand-held refractometers (Eclipse 45–81 and Eclipse 45–82, Bellingham+ Stanley Ltd). It was converted to g∙L^−1^ using a conversion table (Kearns and Inouye 1993). When nectar volumes were too small, samples were diluted with MilliQ water (Millipore Corporation) before measurement. In these cases, the nectar concentration in the flower (C1) was calculated from the dilution equation C1V1 = C2V2, where V1 is the nectar volume sampled in the flower, C2 is the diluted nectar concentration and V2 is the diluted nectar volume. Refractometers were calibrated with a 30 % sucrose solution at 20 °C. Because *S. alba* nectar contains other sugars than sucrose (Vattala *et al.* 2006), concentration measurements reported in this study correspond to sucrose equivalent. However, for the sake of brevity, ‘sucrose’ will refer to ‘sucrose equivalent’ in the following. Refractometer measurements were corrected according to air temperature when necessary with a correction table provided with the refractometer. From all these measurements, we studied (i) traits involved in the attractiveness period of *S. alba* (i.e. time to first open flower and flowering duration) as well as (ii) floral traits involved in plant attractiveness to pollinators. These floral traits have been studied either at the plot level or at the plant level as we assumed that pollinators may respond to plant traits both at long (i.e. detecting attractive plants in the environment, outside from plots) and short distance (i.e. choosing the most attractive plant(s) in a close environment, in a plot); see Table 1 and ‘Pollinator visitation’ for more details.

**Table 1.** Summary of plant–pollinator variables. All variables are affiliated to either plant attractiveness or pollinator visitation. These variables were chosen and classified according to the distance at which they may play a role in attractiveness (i.e. long versus short distance) and were either studied at the plot or the plant level (see sections ‘Attractiveness traits of *S. alba*’ and ‘Pollinator visitation’).

Traits category	Variables	Range of influence or response	Study level
Plant attractiveness	Probability of displaying flowers at plot level	Long-distance attractiveness	Plot
	Plot floral display size		
	Plant probability of displaying flowers	Short-distance attractiveness	Plant
	Plant floral display size		
	Flower size		
	Nectar volume		
	Nectar concentration		
Pollinator visitation	Time to first visit on a plot	Response to long-distance attractiveness	Plot
	Probability that a pollinator visited a plot		
	Number of visits per plot (repetition of visits on close conspecific plants)	Response to short-distance attractiveness	Plot
	Probability that a pollinator visited a plant		Plant
	Number of visits per plant (repetition of visits on a same plant)		

### Pollinator visitation

Every day suitable for pollinator foraging (i.e. no rain, wind speed < 5 m∙s^−1^ and air temperature > 15 °C), plots with flowered plants (and with newly open flowers) were selected for flower-visitor observation sessions. Sessions occurred between 8:30 a.m. and 4:30 p.m. Over the course of the experiment, each plot was observed 5–15 times (9 times on average); these differences come from the fact that plots did not always have plants with newly open flowers. All plots were observed on morning and afternoon sessions to limit the influence of time of day. An observation session was conducted as follows: after floral and nectar trait measurements, the nylon mesh cage was lifted, allowing flower visitors to visit the plot. As observations were made to the naked eye, flower visitors were only identified to easily recognizable morphogroups: honeybees (*Apis mellifera*), solitary bees, bumblebees (*Bombus* spp.), butterflies, syrphids, other dipteran and beetles. An observation session began with the first visit on a plant and lasted for 20 min. Visits were followed at the plant level. Visits per flower as well as visits made by the same flower-visitor individual or by different individuals of the same morphogroup were not discriminated. At the end of the session, the nylon mesh cage was put back and any remaining flower visitor was removed from the cage. The time between the lifting of the nylon cage and the first visit was also recorded (the time to first visit). We waited up to 30 min to observe a first visit and if there was no visit after 30 min we ended the observation session. We assumed that our pollinator visitation variables were the reflection of plant attractiveness either at long (plant detection, away from the plots) or at short distance (plant choice in a plot). For plant detection at long distance, pollinators can use contrasting colours, especially from the environment background (van der Kooi *et al.* 2019) and may rely on the ‘mass effect’ of flowered plots (e.g. the aggregation of flowered plants in a plot, Dafni *et al.* 1997) rather than sparse individuals to locate potential attractive plants. Spaethe *et al.* (2001) showed that there was a correlation between the search time and the colour contrast of target plants with their green background, especially for large flowers. Consequently, we hypothesized that the greater the plot floral display size, the greater the background contrast, the faster the visit. Thus, the time to first visit was studied as a response to plot floral display size, reflecting the long-distance interaction between plants and pollinators. Likewise, the probability that a pollinator visited a plot was considered the reflection of long-distance interactions as sometimes not even one flying pollinator approached poorly flowered plots (personal observations). At short distance, pollinators should rely on plant floral traits (e.g. plant floral display size, flower size) as they can be related to plant rewards (e.g. nectar–corolla length correlation, Gómez *et al.* 2008). Thus, the probability that a pollinator visited a plant and the number of visits per plant was studied as a response to short-distance detectable plant traits. Finally, pollinators will tend to stay on a patch as long as it offers sufficient rewards for a maximized rate of net energy intake (Charnov 1976). If close conspecific plants are attractive and rewarding, we assumed that pollinators were more likely to explore plants within a plot than leaving it. Thus, the number of visits per plot was also considered as a response of pollinators at short distance, reflecting pollinators’ decision to stay on a plot.

Meteorological parameters (air temperature, air humidity, solar irradiance and wind speed) that may affect flower-visitor foraging behaviour were obtained from a weather station (Campbell CR23X equipped with temperature, hygrometer, solar radition and wind sensors) located at a distance of 50–80 m from the experimental plots.

### Fecundity of *S. alba*

At the end of the flowering period, fruits were collected in all plots. Due to fruit herbivory and a late harvest, we were not able to estimate the total number of fruits produced per plant. However, for each plant and each fruit, seeds were counted, weighed on a microbalance (Ohaus Pioneer™ 0.0001 g precision) and their diameter was measured with a digital calliper by taking the hilum (former attachment of the ovule to the ovary) as a reference point. For each fruit, the numbers of unfertilized ovules and aborted seeds were also counted. Based on these numbers, a ‘proportion of viable seeds’ was calculated by dividing the number of viable seeds by the total number of ovules (i.e. sum of viable seeds, aborted seeds and unfertilized ovules). This proportion ranged from 0 to 1 (from no ovule to all ovules turning into viable seeds).

### Data analysis

For an overview of data collection, **see****Supporting Information—Table S1**.

All statistical analyses were performed with R 3.5 (R Core Team 2020). Continuous data (i.e. plant stem height, flowering duration, flower size and nectar volume) were analysed using linear mixed-effects models (LMMs hereafter, *nlme* package, Pinheiro *et al.* 2020) in which competition treatment was set as a fixed effect. Data were transformed when needed to restore normality (plant height was log-transformed, flower size was square-transformed, whereas flower duration and nectar volume were square root-transformed). The association between floral traits and visits was tested using Spearman rank correlation tests.

Count data (floral display size, number of visits and seed number) were analysed using generalized linear mixed-effects models (GLMMs hereafter, *lme4* package, Bates *et al.* 2015) with competition treatment set as a fixed effect. In the case of count data, because our data could contain lots of zeros (e.g. plants that had no flowers or that did not receive visits during the observation sessions), we carried out analyses following a two-step process (Zuur *et al.* 2009). First, a binomial model was fitted to the data to test whether the treatment influenced the proportion of positive counts. Then, a Poisson model was fitted to non-null counts only to test whether the treatment had an effect on count values. For all GLMMs, treatment effect was tested by comparing the fitted model to an intercept-only model and applying a likelihood-ratio test (LRT).

In both LMMs and GLMMs some studied variables came from repeated measures at the plant level (e.g. floral display size, flower size, nectar traits, number of visits), at the plot level (e.g. floral display size, number of visits) and across time. This pseudo-replication was controlled in models by setting the plot and date or the date only (depending on the level at which the variable was studied) as random effects. When necessary, for Poisson models, an observation level random effect was considered to model overdispersion in the data (Harrison 2014).

Survival models (or ‘time-to-event’ analysis) were performed to test the effect of the competition treatment on the time to first open flower for a plant and on the time to first visit on a plot (log-rank test, *survival* package, Therneau 2020). Here, these analyses calculate the probability for an individual (the plant or the plot) to not experience an event across time (here flowering or visitation), depending on competition treatment.

Finally, the effect of competition on plant biomass, nectar concentration, seed size, seed weight and proportion of viable seeds were analysed using Wilcoxon rank sum tests due to non-normality of the data.

As we performed multiple statistical tests on the data (*N* = 24), all *P*-values reported in the Results section have been corrected with the false discovery rate (FDR) correction type (Benjamini and Hochberg 1995; Pike 2011), using the *p.adjust* function in R.

## Results

From 12 June to 11 September 2014, 141 flowers were sampled for size and nectar measurements (C− = 106, C+ = 35). Seventy-two behavioural observations sessions (among which 62 led to visits) were run between 18 June 2014 and 31 July 2014, corresponding to 1440 min of observation (C− = 38 sessions, 760 min and C+ = 34 sessions, 680 min).

### Consequences of below-ground competition on plant growth and biomass

After 82 days of growth, plants in the C+ treatment were 44 % smaller than plants in the C− treatment in average (*F*_1, 5_ = 18.50, *P* = 0.01; see Table 2), indicating that the presence of a competitor had a negative effect on the growth of *S. alba*. The same pattern was observed for above- and below-ground biomass measured at the end of the experiment (respectively, 69 % and 75 % less biomass in C+ compared to C−; *W*_above_ = 786, *P*_above_ = 1.54e-09 and *W*_below_ = 664, *P*_below_ = 1.40e-07; see Table 2).

**Table 2.** Summary of all studied variables, associated means (± SE) according to treatment (C−: without competition; C+: with competition), statistical methods, statistic values and corrected *P*-values (FDR method). Asterisks show corrected *P*-values < 0.05. Abbreviations for statistical methods: LMM, linear mixed models; GLMM, generalized linear mixed models; Wilcoxon RT, Wilcoxon rank test; SA, survival analysis; Spearman RT, Spearman rank correlation test. Abbreviations for statistic values: *F*, Fisher statistic; *W*, Wilcoxon statistic; χ ^2^, chi-squared statistic; LRT, likelihood ratio statistic. Subscript numbers refer to degrees of freedom.

	Mean (± SE)				
	C−:	C+:	Statistical method	Statistic value	Corrected *P*-value
Vegetative traits (at the end of the experiment)					
Final plant height (cm)	91.30 ± 4.28	50.77 ± 5.38	LMM	*F*_1, 5_ = 18.50	*P* = 0.01*
Above-ground biomass (g)	1.44 ± 0.13	0.44 ± 0.24	Wilcoxon RT	*W* = 786	*P* = 1.54e-09*
Below-ground biomass (g)	0.08 ± 0.01	0.02 ± 0.01	Wilcoxon RT	*W* = 664	*P* = 1.40e-07*
Attractiveness period					
Survival analysis on time to first open flower	–	–	SA	$\chi_{1}^{2}$ = 4.6	*P* = 0.05
Flowering duration (days)	40.35 ± 3.42	19.91 ± 3.91	LMM	*F*_1, 6_ = 7.37	*P* = 0.05
Attractiveness traits and pollinator response—long distance					
Plot probability of displaying flowers	0.81	0.51	GLMM	LRT = 64.33	*P* = 1.26e-14*
Plot floral display size (plot FDS)	49.31 ± 3.39	11.42 ± 1.58	GLMM	LRT = 8.79	*P* = 6.06e-03*
Probability that a pollinator visited a plot	1	0.73	GLMM	LRT = 15.10	*P* = 3.50e-04*
Survival analysis on time to first visit	–	–	SA	$\chi_{1}^{2}$ = 13.2	*P* = 7.20e-04*
Correlation plot FDS − time to first visit (ρ = −0.46)	–	–	Spearman RT	*S* = 4.52e4	*P* = 7.20e-04*
Attractiveness traits and pollinator response—short distance					
Plant probability of displaying flowers	0.38	0.10	GLMM	LRT = 13.28	*P* = 7.20e-04*
Plant floral display size (plant FDS)	9.96 ± 0.3	5.33 ± 0.69	GLMM	LRT = 171.95	*P* = 5.28e-15*
Flower size (mm)	13.81 ± 0.23	11.64 ± 0.28	LMM	*F*_1, 6_ = 17.94	*P* = 9.43e-03*
Nectar volume (µL)/fleur	0.114 ± 0.01	0.115 ± 0.02	LMM	*F*_1, 6_ = 0.62	*P* = 0.58
Nectar concentration (g of sucrose per L)/fleur	515.56 ± 70.54	439.08 ± 74.60	Wilcoxon RT	*W* = 1133	*P* = 0.77
Probability that a pollinator visited a plant	0.72	0.70	GLMM	LRT = 0.06	*P* = 0.80
Number of visits/flowered plant	13.67 ± 1.15	15.05 ± 3.19	GLMM	LRT = 0.11	*P* = 0.77
Correlation plant FDS − number of visit/plant (ρ = 0.24)	–	–	Spearman RT	*S* = 3.50e6	*P* = 1.26e-04*
Number of visits/flowered plot	62.97 ± 9.50	26.33 ± 6.15	GLMM	LRT = 10.00	*P* = 3.40e-03*
Correlation plot FDS − number of visit/plot (ρ = 0.57)	–	–	Spearman RT	*S* = 2.48e4	*P* = 1.65e-06*
Consequence on plant seeds					
Seed size (mm)	2.36 ± 0.02	2.32 ± 0.03	Wilcoxon RT	*W* = 8388	*P* = 0.50
Seed weight (g)	5.46e-03 ± 1.08e-04	5.64e-03 ± 2e-04	Wilcoxon RT	*W* = 7868	*P* = 0.77
Viable seed number/fruit	2.32 ± 0.11	2.14 ± 0.18	GLMM	LRT = 0.32	*P* = 0.69
Proportion of viable seeds per fruit	0.69 ± 0.03	0.46 ± 0.04	Wilcoxon RT	*W* = 997	*P* = 9.43e-03*

### Consequences of below-ground competition on attractiveness period of *S. alba*

Plants from both treatments started flowering at the same time, ca. 43 days after the start of the experiment **[see****Supporting Information—Fig. S2****]**, with more plants in bloom in the C+ treatment (18 plants) compared to the C− treatment (10 plants). At the end of the experiment, all plants from the C− treatment had produced at least one flower while 19 % of plants in the C+ treatment did not bloom at all **[see****Supporting Information—Fig. S2****]**. Nevertheless, the survival analysis on time to first open flower detected a tendency but no significant difference between treatments (log-rank test, χ ^2^ = 4.6, *P* = 0.05; Table 2). Likewise, there was a tendency but no significant difference in flowering duration between treatments despite a flowering span almost twice as short for plants in the C+ treatment (*F* = 7.37, *P* = 0.05; Table 2).

### Consequences of below-ground competition on attractiveness of *S. alba—*long distance

#### Attractiveness traits.

**Figure 1.**
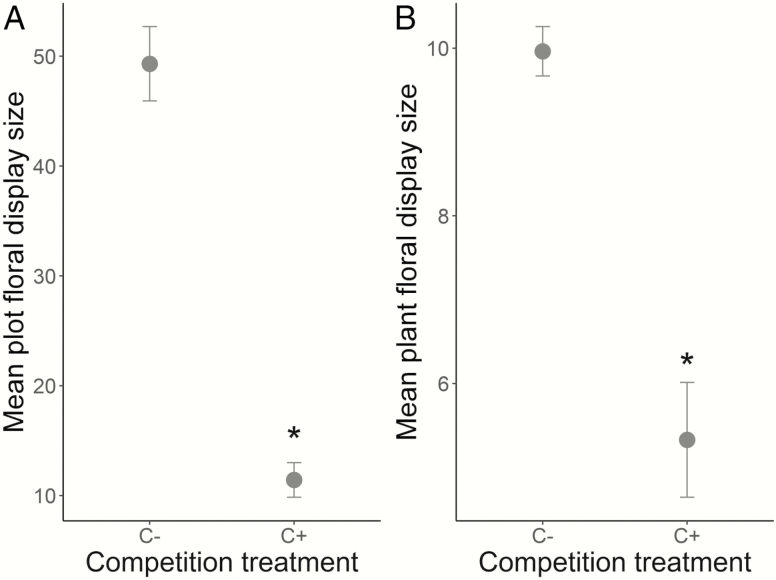
Mean floral display size (± SE) of *S. alba* without (C−) and with competition (C+) at (A) the plot and (B) the plant level. A star represents a significant difference (*P*_plot_ = 6.06e-3, *P*_plant_ = 5.28e-15, GLMM).

In the C+ treatment, plots had a 37 % lower probability of displaying flowers (LRT = 64.33, *P* = 1.26e-14; Table 2) and had a floral display size 77 % smaller, compared to C− plots (LRT = 8.79, *P* = 6.06e-03; Table 2; Fig. 1A).

#### Pollinators’ visitation.

**Figure 2.**
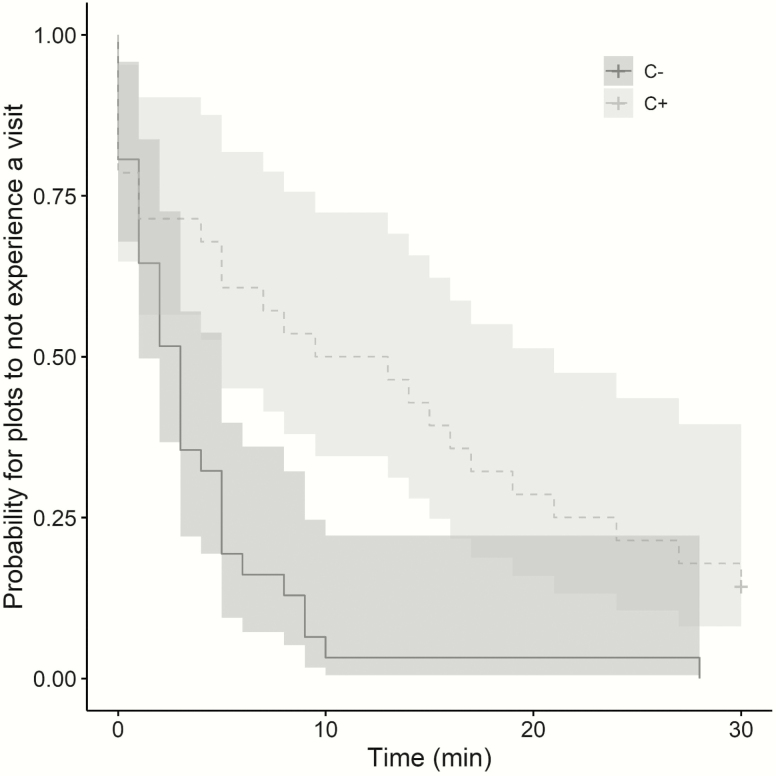
Survival curves representing the time to first visit on plots (or the probability for plots of *S. alba* to not experience a visit across time), without (C−) and with competition (C+). It can be interpreted as the proportion of plots yet to be visited for a given time or the time it takes for a given proportion of plots to experience a visit, across time. The time represents the number of minutes since nylon mesh cage removal. Grey areas around the curves correspond to the 95 % confidence interval of a Kaplan–Meier test (*P* = 7.20e-04).

The probability that a pollinator visited a plot was significantly lower in condition of competition with, some plots that were never visited (LRT = 15.10, *P* = 3.05e-04; Table 2). The survival analysis on time to first visit also revealed a significant difference between treatments (χ ^2^ = 13.2, *P* = 7.20e-04). The first visit on a plot occurred in <10 min after the removal of the nylon mesh cage for most C− plots (96.8 %), whereas only 50 % of C+ plots received a first visit during this time (Fig. 2). All observation rounds on C− plots led to at least one visit from a pollinator while 12.1 % of observation rounds on C+ plots did not lead to any visit (Fig. 2). Time to first visit on a plot was significantly negatively correlated to plot floral display size (*S* = 4.52e4, *P* = 7.20e-04, ρ = −0.46; Table 2). When the plot floral display size increased, it took less time for plots to receive a visit.

### Consequences of below-ground competition on attractiveness of *S. alba*—short distance

#### Attractiveness traits.

In C+ treatment, *S. alba* plants had a 72 % smaller probability of displaying flowers (LRT = 13.28, *P* = 7.20e-04; Table 2) and a floral display size 47 % smaller than in C− plots (LRT = 171.95, *P* = 5.28e-15; Table 2; Fig. 1B). In addition, plants of *S. alba* in the C+ treatment produced significantly smaller flowers than plants in the C− treatment (a 16 % decrease in size in C+ plots, *F*_1, 6_ = 17.94, *P* = 9.43e-03; Table 2). Concerning rewards, the daily nectar volume and the daily nectar concentration were not affected by the competition treatment (both *P* > 0.05; see Table 2).

#### Pollinators’ visitation.

**Figures 3.**
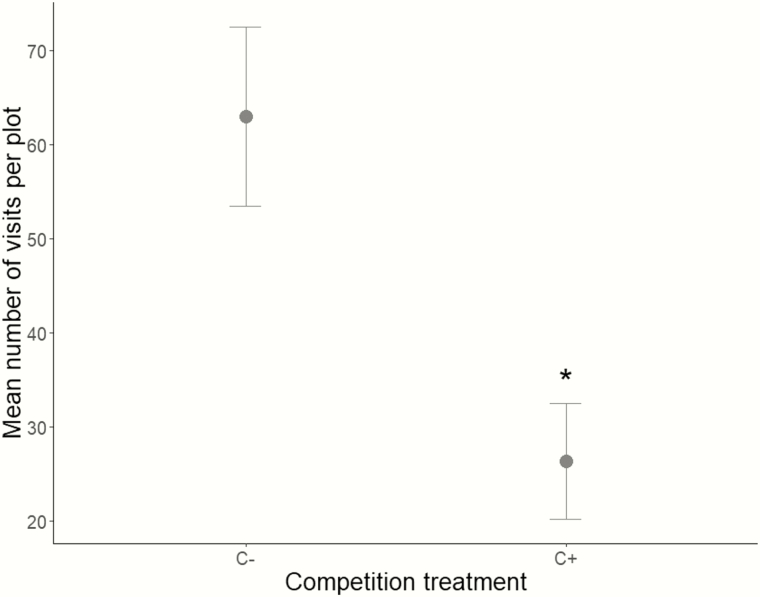
Mean number of pollinator visits per plot (± SE) without (C−) and with competition (C+). A star represents a significant difference (*P* = 3.40e-03, GLMM).

As 97.7 % of visits were made by solitary bees, hoverflies and bumblebees, we focused our analyses on these three flower-visitor morphogroups. Neither the probability that a pollinator visited a plant, nor the number of visits received per plant of *S. alba* differed between treatments (both *P* > 0.05; see Table 2). However, there was a correlation between plant floral display size and the number of visits received by *S. alba* plants (*S* = 3.50e6, *P* = 1.26e-04, ρ = 0.24; Table 2). When summing the number of visits per plant on all plants per plot, competition treatment had a negative influence with a decrease of 58 % compared to C− plots (LRT = 10.00, *P* = 3.40e-03; Table 2; Fig. 3). Correlation between the number of visits received per plot and plot floral display size was also significant (*S* = 2.48e4, *P* = 1.65e-06, ρ = 0.57; Table 2).

### Consequences on fecundity of *S. alba*

**Figure 4.**
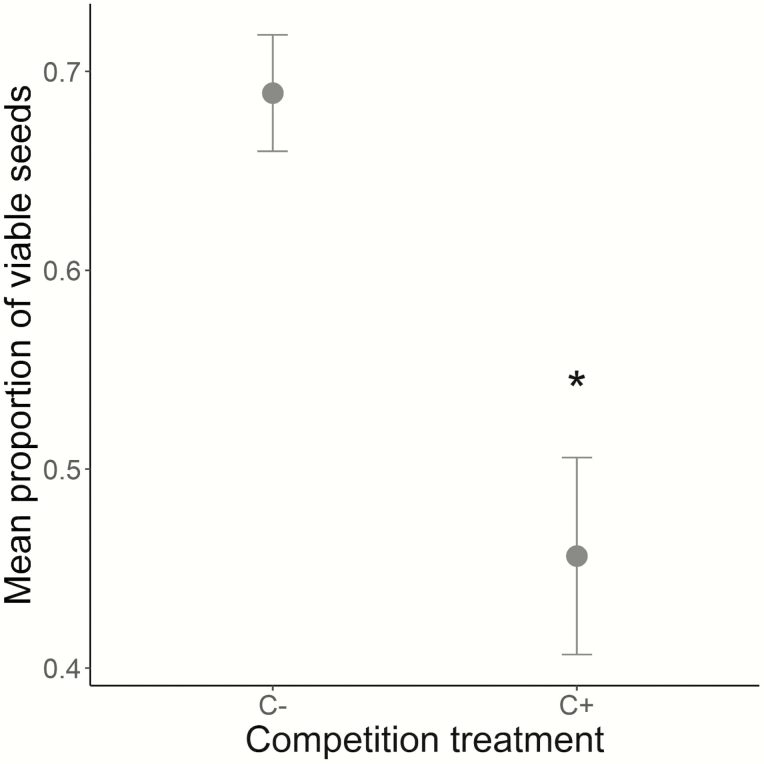
Mean proportion of viable seeds (± SE) of *S. alba* without (C−) and with (C+) below-ground competition. A star represents a significant difference (*P* = 9.43e-03, Wilcoxon rank test).

At the end of the experiment, a total of 199 fruits were collected (C+ = 48 from 23 plants, C− = 151 from 32 plants). There was no influence of the competition treatment on viable seed number per fruit, seed size, seed weight (all *P* > 0.05; see Table 2). However, in below-ground competition with *H. lanatus*, plants of *S. alba* displayed fruits with a 30 % lower proportion of viable seeds (*W* = 997, *P* = 9.43e-03; Fig. 4; Table 2).

## Discussion

In this study, our objective was to test if the presence of a competitor plant species could alter interactions between an insect-pollinated species and wild pollinators through below-ground competition. Our results suggest that (i) competition induced by the presence of a competitor species can negatively impact floral traits involved in the attractiveness of *S. alba* and (ii) that these modifications translate to differences in pollinator visitation. Especially, the long-distance detection of a flowered plot by pollinators tended to be altered in context of below-ground competition. Moreover, pollinators tended to do less visits on plots submitted to competition. Our results on *S. alba* fecundity, however, deserve more caution.

### Vegetative traits of *S. alba* and below-ground competition

Height and biomass (both below-ground and above-ground) of *S. alba* decreased when its roots were not isolated from the roots of *H. lanatus*, the competitor plant. By measuring plant biomass, we assessed the competition outcome rather than the competition itself (Trinder *et al.* 2012). Nevertheless, Flacher *et al.* (2015, 2017) showed that *H. lanatus* plants can display a large and dense root system that can lead to a reduction of their neighbour’s biomass. Moreover, plant size and biomass can lead to some advantages especially for the occupation of soil space and nutrient acquisition (Berntson and Wayne 2000; Raynaud and Leadley 2004; Gurevitch *et al.* 2006). Thus, a decrease in plant height and biomass, here in condition of competition, suggests that one mechanism by which competition could alter plant traits such as floral traits involved in plant attractiveness could be resource depletion.

### Influence of below-ground competition on floral traits involved in attractiveness of *S. alba* at long and short distance

Several floral traits involved in the attractiveness of *S. alba* at long (i.e. plot probability of displaying flowers, plot floral display size) and short (i.e. plant probability of displaying flowers, plant floral display size, flower size) distance were negatively influenced by the competition treatment. This has been observed in previous studies, especially at the plant level (Baude *et al.* 2011; Partzsch and Bachmann 2011; Flacher *et al.* 2015, 2017) probably through soil resource depletion as floral traits can be sensitive to soil resource availability (Muñoz *et al.* 2005; Burkle and Irwin 2009). In contrast, reward traits such as nectar volume and concentration remained constant regardless of the competitive context. Nectar volume and concentration in *S. alba* were variables as reported within some plant species (Nicolson *et al.* 2007). Detecting an effect of below-ground competition might need a greater sampling effort. Nevertheless, some studies showed that nectar traits are not always influenced by the modification of available resources (David *et al.* 2019). Nectar is a costly production (Southwick 1984) and reward quantity and quality can have a strong influence on pollinator behaviour (in relation to nutritional needs, Dafni 1992; Goulson 2003) that can in turn influence pollination efficiency (Cayenne Engel and Irwin 2003). Therefore, there might be a trade-off in the production of traits involved in attractiveness to pollinators with constant reward production at the expense of flower production. Previous experiments have found that in some insect-pollinated plants species, nectar quantity and quality were reduced in the presence of a competitor plant when integrated across the whole flowering season (e.g. total nectar volume produced by a plant across flowering season; Baude *et al.* 2011; Flacher *et al.* 2015). However, they were not affected at the flower level when measured daily (Flacher *et al.* 2015). Therefore, rewards could be affected by competition only through the production of flowers. Such integrated variables are of interest to study plant–pollinator interactions as they can be associated to pollinator visitation (Leiss and Klinkhamer 2005).

### Influence of below-ground competition on pollinator visitation at long and short distance in link to attractiveness traits

Below-ground competition had a negative influence on pollinator response at long and short distance. Especially, changes in floral display size may have played a key role here as it can influence pollinator behaviour at different distances (Oberrath and Böhning Gaese 1999). For a given time, the proportion of C+ plots that received a visit across time was lower or in other words it took more time for a given proportion of C+ plots to be visited. Moreover, all C− plots were visited while some C+ plots received no visit. In addition, the more flowers a plot contained, the less time it took for a given proportion of plots to be visited. At the plot level, the greater the floral display size is, the more the plot may be visible to pollinators (through floral aggregation, Dafni *et al.* 1997; Hempel de Ibarra *et al.* 2015) and the more it may attract them from long distance. Once in a plot (i.e. at short distance), we expected pollinators to do more visits per plants grown without competition. Nevertheless, we only observed more visits when summed on all plants within a plot. In plots without competition, pollinators were surrounded by more flowered plants with, in addition, large floral display size. Attractive close conspecific plants may have encouraged pollinators to stay on a plot and visit several plants as suggested by pollinator foraging economics as long as they met adequate rewards (Charnov 1976). The decrease in the number of visits in condition of competition might be only due to C+ plots displaying fewer flowered plants, with, in addition smaller floral display size. More generally, there was a positive correlation between the number of visits on a plant and the plant floral display size. This relation suggests that, once in a plot, a pollinator may preferentially visit a plant that has the greater number of open flower as observed in several studies (Conner and Rush 1996; Mitchell *et al.* 2004; Grindeland *et al.* 2005; Miyake and Sakai 2005) whatever the competition treatment. Floral display size can be a reliable cue for pollinators to assess if a plant is rewarding as more flowers may indicate more available rewards (Makino and Sakai 2007; Flacher *et al.* 2015). However, this study focused on flowers and nectar while other plant traits involved in pollinator behaviour could have been modulated by below-ground interactions as well. For instance, pollen quantity and quality, the production of secondary metabolites in nectar (see David *et al.* 2019 for a review) or of volatiles compounds (Veromann *et al.* 2013) can also be sensitive to soil resource availability. Likewise, non-floral traits such as plant height can influence pollinator behaviour (Richardson *et al.* 2016) and can be modulated by competitive interaction. It is important to note that these traits could also act on pollinator response to below-ground competition at both distances.

### Influence of below-ground competition on plant fecundity in link to attractiveness traits

We expected that below-ground competition would have an influence on plant fecundity traits both directly from a decrease in available soil resources and indirectly from modification of pollinator visitations. Below-ground competition reduced the proportion of viable seeds of *S. alba.* As *S. alba* plants produced fruits containing the same number of viable seeds in both competition treatments, this decrease is likely due to a difference in the total number of produced ovules (i.e. the addition of viable seeds, aborted seeds and unfertilized ovules). Indeed, in the competition treatment, plants displayed fruits with a higher number of produced ovules. This suggests that pollinator visits had a limited influence on the proportion of viable seeds (e.g. Burkle and Irwin 2009) compared to the availability of soil resources. Here, a greater number of ovules may be the result of a resource allocation trade-off with other reproductive traits such as floral display size. Nevertheless, these supplementary ovules seem to have not been fertilized. Some studies demonstrated that the number of visits at the plant level could influence its seed set (Geslin *et al.* 2017) but here *S. alba* plants received the same number of visits between treatments. The number of visits at the flower level would have been more informative. When dividing the number of visits per plant by the number of flowers per plant, plants of *S. alba* subjected to competition received more visits per flower but probably just because of fewer available flowers. These flowers may still have not received a sufficient amount of pollen (again, fewer flowers to receive pollen from, potential alteration of pollen production) to fertilize this greater number of ovules, leading to some pollen limitation (Willmer 2011).

Concerning seed morphometrical traits (i.e. size and weight), we did not observe any differences depending on the competition treatment. Seed size constancy has sometimes been interpreted as the result of developmental and/or morphological constraints (Silvertown 1989) and researchers have suggested that the high inter- and intra-plant variation among plant species could lead to apparent constancy in mean seed size (Fenner 1985). Even though this was observed for very large samples, we did observe intra- and inter-plant variations at both competition levels in our study, that may explain the absence of an overall competition effect. Overall, we should mention that due to late harvest and fruit herbivory, these fecundity traits were assessed on a subset of the total produced seeds. For the same reason, we were not able to test for any influence of below-ground competition on fruit production and morphometry. This may have interfered with our estimation of plant fecundity in a context of below-ground competition, and potentially on our ability to discriminate the direct influence of competition (through modification of soil resources) from its indirect effect (through modifications of pollinator visitation).

### Conclusions and perspectives

Altogether, this study suggests that, in a plant mixture, the presence of any competitor plant may alter the long- and short-distance pollinator attractiveness of its insect-pollinated neighbours through below-ground interactions. Future studies on plant–pollinators interactions may benefit from considering the whole plant community. This is of substantial importance for (i) fundamental knowledge on below-ground and above-ground ecological linkages as well as (ii) developing appropriate management practices for plant and pollinator populations (e.g. grassland restoration, seed mixtures). To go further, future research may investigate community issues such as the combined effect of plant competition and below-ground organisms on plant–pollinator interactions through plant traits.

## Supporting Information

The following additional information is available in the online version of this article—

**Figure S1.** Initial experimental design.

**Figure S2.** Survival curves representing the ‘time to first open flower’ (or the probability for plants of *Sinapis alba* to not experience flowering across time), without (C−) and with competition (C+).

**Table S1.** Summary of all measured variables and sampling effort.

plaa022_suppl_Supplementary_Material

## Data

Data and R script are available on ZENODO with the following doi: https://doi.org/10.5281/zenodo.3833619
