# Supplementary material for: Below-ground competition alters attractiveness of an insect-pollinated plant to pollinators
Source: AoB Plants. 2020 Jun 12;12(4):plaa022. doi: 10.1093/aobpla/plaa022 (PMC7384324; doi:10.1093/aobpla/plaa022)
Supplement: plaa022_suppl_Supplementary_Material [file plaa022_suppl_supplementary_material.pdf]

Supporting information

Figure S1: Initial experimental design. There were 4 treatments: *S. alba* roots subjected to belowground competition (1), *E. plantagineum* roots subjected to belowground competition (2), no focal roots subjected to belowground competition (3) and both *S. alba* and *E. plantagineum* roots subjected to belowground competition (4). Because of *Echium* issues (*E. plantagineum* seeds being of *E. vulgare*), we focused only on 2 treatments for floral traits and pollinator response. We chose treatments 2 and 4 as it limited the number of plastics pots in plots and thus, potential modification of soil structure and water flow, inherent issues of this type of design. As *H. lanatus* is known to outcompete Echium species (Flacher *et al.* 2015) we assumed the competition was mainly due to this plant roots. In the end of the experiment, this was confirmed thanks to measures of biomass and height of *S. alba* plants : we found no differences between treatments 1 and 4 as well as between treatments 2 and 3, meaning that *Echium* roots had no major influence and that belowground competition was mainly due to *H.lanatus* roots.

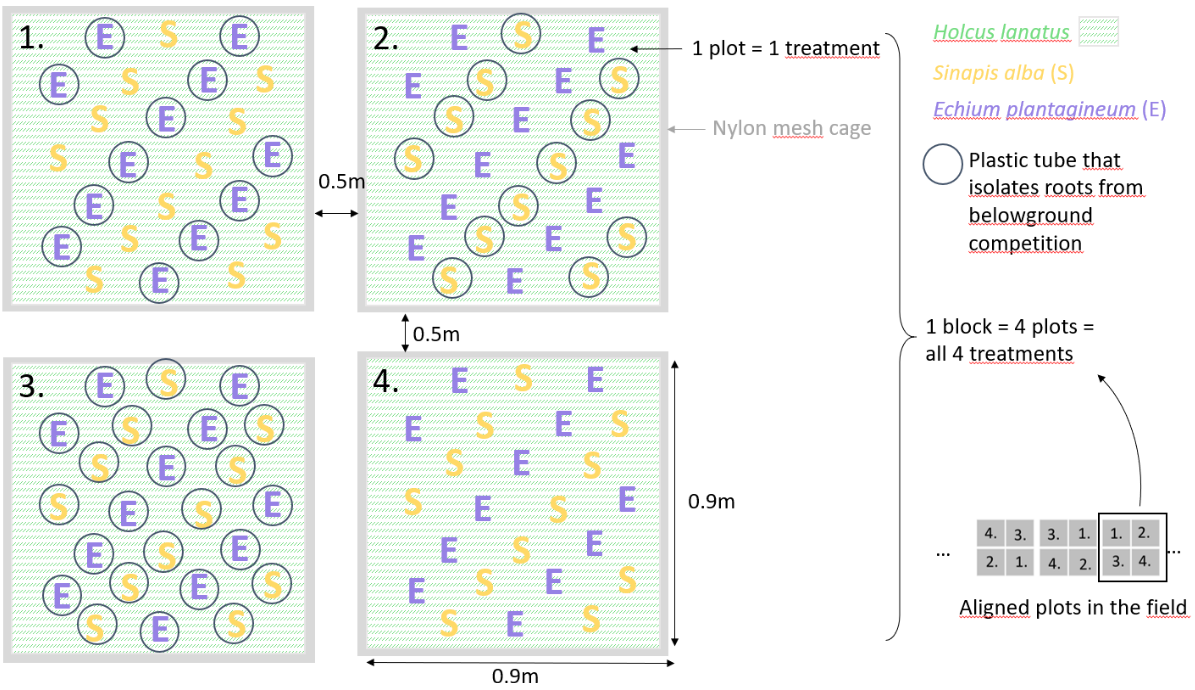

Figure S2 : Survival curves representing the “time to first open flower” (or the probability for plants of *S. alba* to not experience flowering across time), without (C-) and with competition (C+). It can be interpreted as the proportion of plants yet to be flowering for a given time or the time it takes for a given proportion of plants to experience flowering, across time. The time represents the number of days after the start of the experiment. Grey areas around the curves correspond to the 95% confidence interval of a Kaplan-Meier test. (P=0.05).

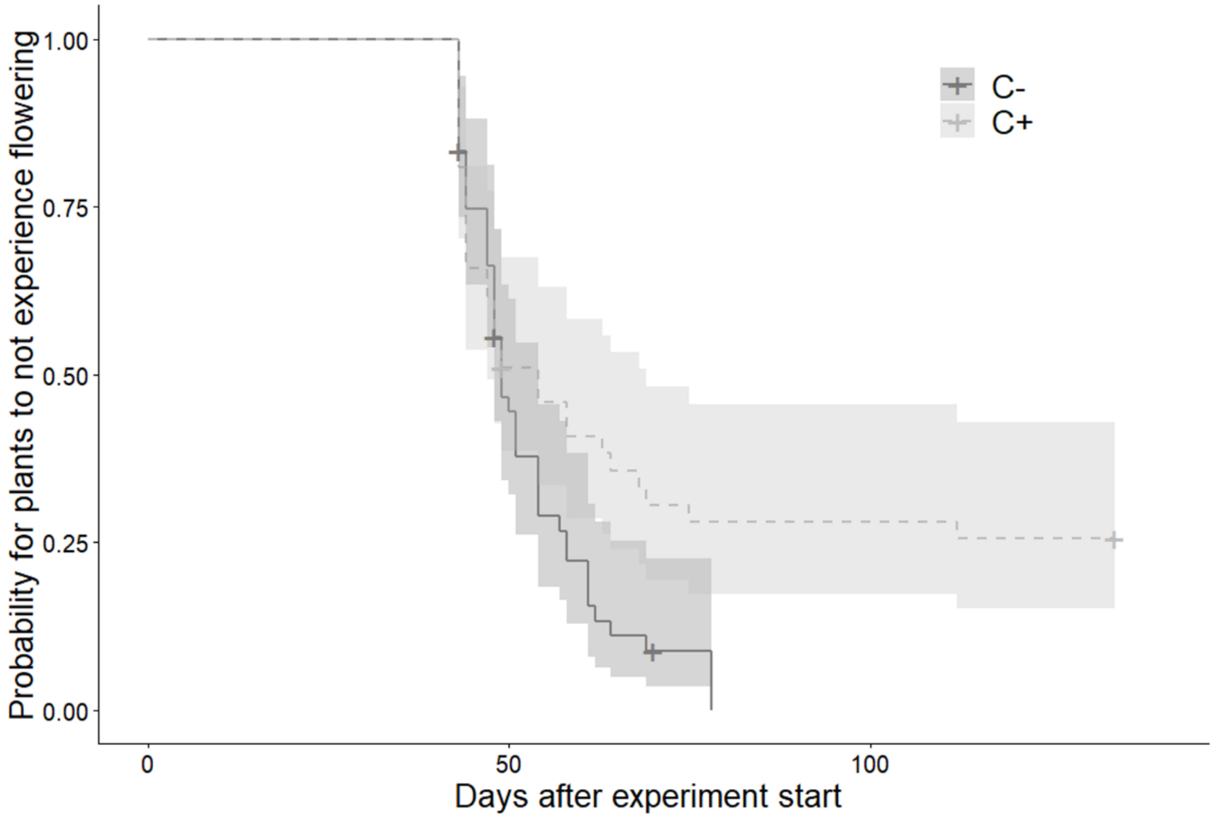

Table S1: Summary of all measured variables and sampling effort.

	Variable measured in site or inferred from the data	Level at which it is measured / inferred	Number of plot / plant / flowers available for measures or for inference (all treatments)	Sampling frequency (all treatments)
**Vegetative traits**				
Plant height (cm)	Measured	Plant	96 plants in 8 plots	1 date
Aboveground biomass (g)	Measured	Plant	96 plants in 8 plots	1 date
Belowground biomass (g)	Measured	Plant	96 plants in 8 plots	1 date
**Attractiveness period**				
Time to first open flower	Measured	Plant	96 plants in 8 plots	1 date
Flowering duration (days)	Inferred	Plant	96 plants in 8 plots	1 date
**Attractiveness traits and pollinator response – Long distance**				
Plot probability of displaying flowers	Inferred	Plot	8 plots	55 dates
Plot floral display size	Inferred	Plot	8 plots	55 dates
Probability that a pollinator visited a plot	Inferred	Plot	8 plots	72 sessions in 55 dates
Time to first visit	Measured	Plot	8 plots	72 sessions in 55 dates
**Attractiveness traits and pollinator response – Short distance**				
Plant probability of displaying flowers	Inferred	Plant	96 plants in 8 plots	55 dates
Plant floral display size	Measured	Plant	96 plants in 8 plots	55 dates
Flower size (mm)	Measured	Flower	141 sampled flowers on 28 plants in plots	72 sessions in 55 dates
Nectar volume (µL) / fleur	Measured	Flower	141 sampled flowers on 28 plants in plots	72 sessions in 55 dates
Nectar concentration (g of sucrose.L^{-1}) / fleur	Measured	Flower	141 sampled flowers on 28 plants in plots	72 sessions in 55 dates
Probability that a pollinator visited a plant	Inferred	Plant	96 plants in 8 plots	55 dates
Number of visits / plant	Measured	Plant	96 plants in 8 plots	72 sessions in 55 dates
Number of visits / plot	Inferred	Plot	8 plots	72 sessions in 55 dates
**Consequence on seed set**				
Seed size (mm)	Measured	Seed	407 harvested seeds on 55 plants in 8 plots	1 date
Seed weight (g)	Measured	Seed	407 harvested seeds on 55 plants in 8 plots	1 date
Seed number / fruit	Measured	Fruit (flower)	199 harvested fruits on 55 plants in 8 plots	1 date
Proportion of viable seeds	Inferred	Fruit (flower)	199 harvested fruits on 55 plants in 8 plots	1 date
